# Reflections on the trends of suicide in Sri Lanka, 1997–2022: The need for continued vigilance

**DOI:** 10.1371/journal.pgph.0003054

**Published:** 2024-04-17

**Authors:** Piumee Bandara, Prabath Wickrama, Sambasivamoorthy Sivayokan, Duleeka Knipe, Thilini Rajapakse

**Affiliations:** 1 Population Health Sciences, Bristol Medical School, University of Bristol, Bristol, United Kingdom; 2 South Asian Clinical Toxicology Research Collaboration, Faculty of Medicine, University of Peradeniya, Peradeniya, Sri Lanka; 3 Department of Psychiatry, University of Jaffna, Jaffna, Sri Lanka; 4 Department of Psychiatry, Faculty of Medicine, University of Peradeniya, Peradeniya, Sri Lanka; NIMHANS: National Institute of Mental Health and Neuro Sciences, INDIA

## Abstract

Despite reductions in suicide rates in Sri Lanka during the past decades, largely by introduction of national bans on highly hazardous pesticides, the country continues to record a higher than global average rate of suicide. With the changing availability of methods of suicide over time, we aimed to examine the age-standardized suicide rates in Sri Lanka by sex, age, and method between 1997 to 2022 using national police suicide data to identify trends. The rate of suicide in Sri Lanka in 2022 was 27/100,000 and 5/100,000, in males and females respectively, with an overall suicide rate of 15/100,000 population. When considering the previous decades, the overall rate of suicide has declined from 1997 until about 2015, in both sexes, driven by a drop in the numbers of suicides due to pesticide ingestion. In females the overall rates of suicide plateaued around 2015, but in males there has been an upward trend in overall suicide that started in 2016, mostly due to an increase in rates of hanging. Since 2016 rates of suicide by hanging have increased among older males, and young females (17–25 years). Whilst the current suicide rate in Sri Lanka is substantially lower than it was during the 1990s, the upward trend in hanging seen in the last few years, particularly among older men and young women, is of concern. Ongoing monitoring of suicide rates should be a priority during the next few years, to detect and respond to changes as soon as possible. There is an urgent need to address current risk factors for suicide in Sri Lanka, such as significant financial insecurity, unemployment, depression, alcohol misuse, and domestic violence, and to minimize media glamourization of hanging by suicide.

## Introduction

Since the mid-1990s, Sri Lanka has achieved a 70% reduction in suicide, with an estimated 93,000 suicide deaths prevented mostly by the implementation of bans on identified toxic pesticides [1, 2]. Despite this, the suicide rate in Sri Lanka still remains high [3] and with a current rate well above the global average [4]. Since the end of the civil war in 2009, Sri Lanka has undergone many changes and challenges, including urbanization [5], the Easter Sunday bombing attacks in 2019 [6] and most recently the COVID-19 pandemic and associated intermittent lockdowns during 2020–21 [7].

Emerging out of the shadow of the COVID-19 pandemic, the island nation in 2022 experienced a severe economic crisis, which resulted in a nationwide financial crisis, political instability, islandwide power cuts, fuel shortages, and rising food insecurity [8]. In 2022 inflation in Sri Lanka was at 50%. In the context of this turbulent environment, poverty, internal migration, alcohol and substance misuse, depression and domestic violence are likely amplified, all of which are well-documented risk factors for suicide and self-harm [9–14]. Given this ever-changing and complex social and economic environment, it is critical to identify emerging trends in suicide. We aimed to investigate the rate of suicide between 1997 and 2022 by sex, age, and method to reveal emerging trends. The last time there was an investigation on trends, disaggregated in this way, was nearly a decade ago [15].

## Methods

### Setting

Sri Lanka is an island nation situated in the Indian Ocean. A lower middle income country, Sri Lanka has a multi-ethnic population of approximately 22.1 million.

### Data and analysis

Publicly available anonymized data on the annual count of deaths due to suicide disaggregated by age, sex, and method were available for the years 1997 to 2022 and were obtained from the Department of Police, Division of Statistics, Sri Lanka on 24 March 2023 for research purposes. Although suicide is no longer considered a criminal act in Sri Lanka, nationwide statistics on suicide are maintained by the Police Department, hence we obtained the data from the Police. Data were provided by the police grouped by sex and in five-year age groups, except for the two youngest and the oldest groups (8–16, 17–20, 21–25, 26–30, 31–35, 36–40, 41–45, 46–50, 51–55, 56–60, 61+ years). Data for each sex-age group was also provided by 15 categories of suicide method (namely self-poisoning or self-injury via pesticide ingestion, hanging, drowning, firearms, bombs, cutting, fire, train, ingestion of acids, fuel, drugs and other substances, natural plant poisons, jumping from a height, ingestion of illegal drugs, and other, unspecified). Data on age, sex, and method were complete for all suicide deaths that occurred during the study period.

To estimate the age-standardized suicide rates, publicly available mid-year population estimates for 1997 to 2022 by sex and age were obtained from the Registrar General’s Department, Sri Lanka. As previously done, we have corrected the mid-year estimates to smooth out step changes in population estimates around the Census years [1]. Annual suicide counts by sex and age were divided by the mid-year estimates and multiplied by the WHO World Standard Population to calculate the age-standardized suicide rate per 100,000 population. The above listed age groups were collapsed into four broad age categories for ease of interpretation (17–25, 26–35, 36–55 and 55+ years), which have been used in previous analysis of these data [1, 3]. To improve statistical power and explore leading methods of suicide, the methods of suicide were classified into four categories, namely suicide by 1) non-pesticide self-poisoning 2) pesticide self-poisoning only 3) hanging and 4) all other methods. The male: female suicide ratio overall and by age and method were estimated by dividing the male age-standardized suicide rate by the female age-standardized suicide rate. Graphical presentations were used to investigate trends in the rate of suicide by age, sex, and method over the study period. All analyses were conducted in Stata version 17.

### Ethics statement

This study used anonymised publicly available data on suicide deaths routinely collected by the Police Department in Sri Lanka. No information was provided that could identify individual participants.

## Results

**Fig 1:**
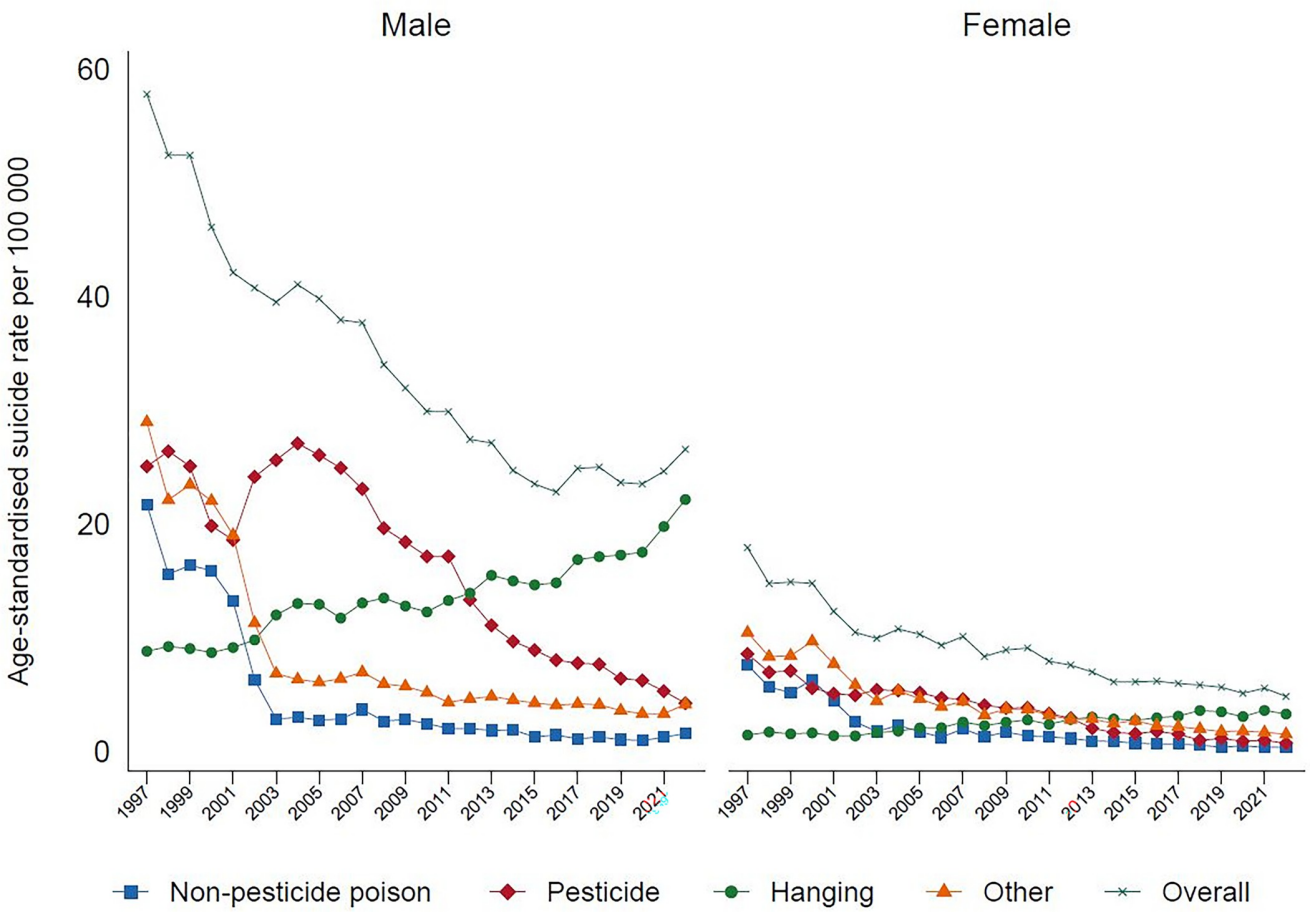
Age-standardized suicide rate by method in males and females in Sri Lanka, 1997–2022.

In 2022, 3406 individuals (83% males) died by suicide, compared to 6418 individuals in 1997 (75% males). The overall age-standardised rate of suicide dropped from 38 per 100,000 in 1997 to 15 per 100,000 in 2022. There were considerable reductions in both male and female suicide rates (Males: from 58 to 27 per 100,000; Females from 18 to 5 per 100,000 in 1997 and 2022 respectively) (Fig 1).

In 2022, among males, the rate of suicide increases by age, with the highest rate among older males (55+ years: 65 per 100,000) (Table 1). Among females, in 2022, the highest suicide rates were found in young females (17–25 years: 10 per 100,000) and over 55 years (9 per 100,000). The female rate of suicide drops to 5 per 100,000 in the two middle age groups (26–35 and 36–55 year olds) (Table 1).

**Table 1:** Sex-specific age-standardised suicide rates and female: Male suicide ratio for 2022 by age group and method, Sri Lanka.

	Female suicide rate	Male suicide rate	Female: male suicide ratio
**Overall**	5 per 100,000	27 per 100,000	1:5
**Age group years**			
17–25	10 per 100,000	28 per 100,000	1:3
26–35	5 per 100,000	30 per 100,000	1:5
36–55	5 per 100,000	35 per 100,000	1:7
56 and over	9 per 100,000	65 per 100,000	1:7
**Method**			
Self-poisoning (non-pesticide)	0.5 per 100,000	2 per 100,000	1:6
Pesticide self-poisoning	1 per 100,000	4 per 100,000	1:4
Hanging	3 per 100,000	22 per 100,000	1:7
Other methods	2 per 100,000	4 per 100,000	1:2

**Fig 2:**
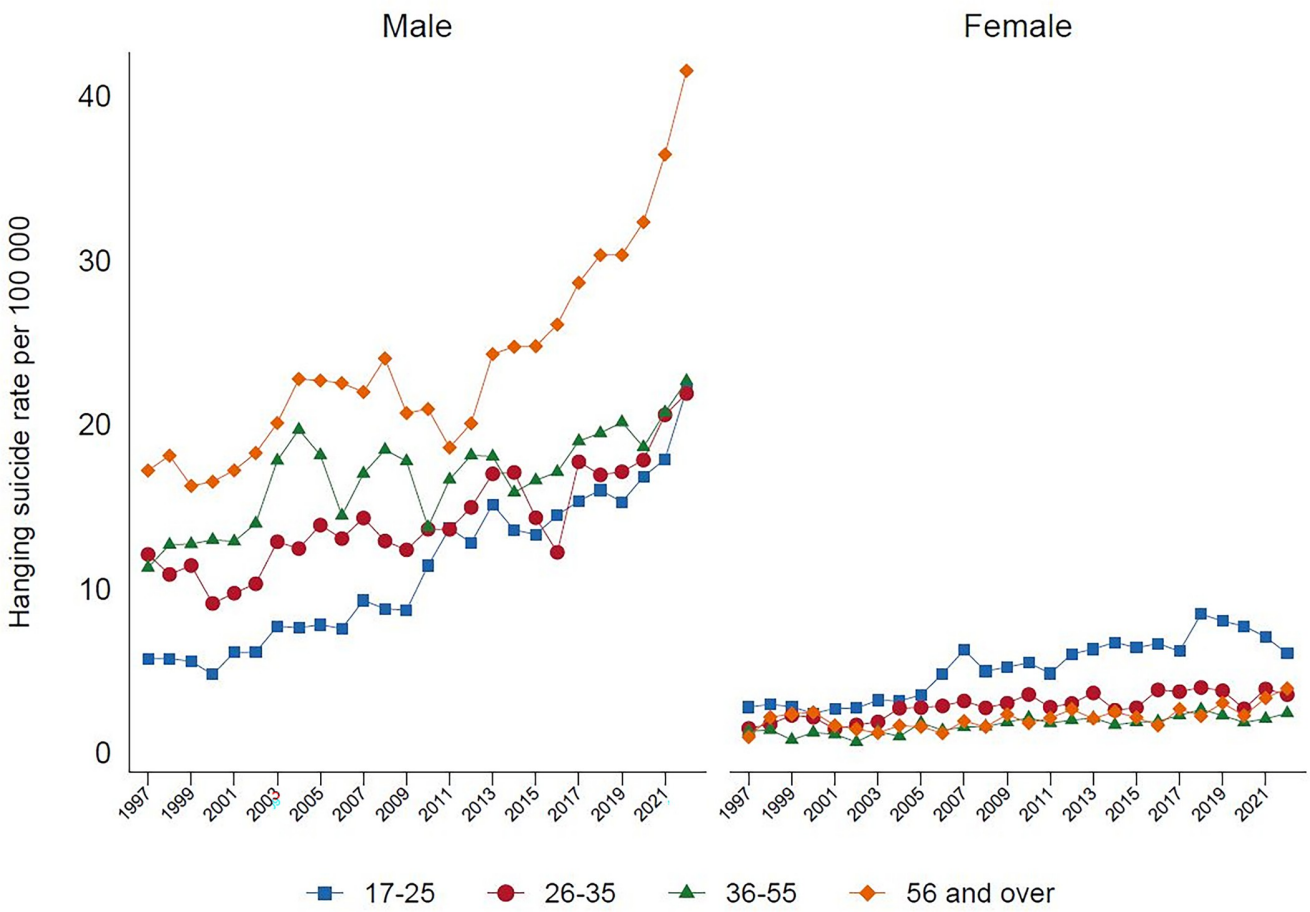
Hanging suicide rate, by age and gender in Sri Lanka, 1997–2022.

**Fig 3:**
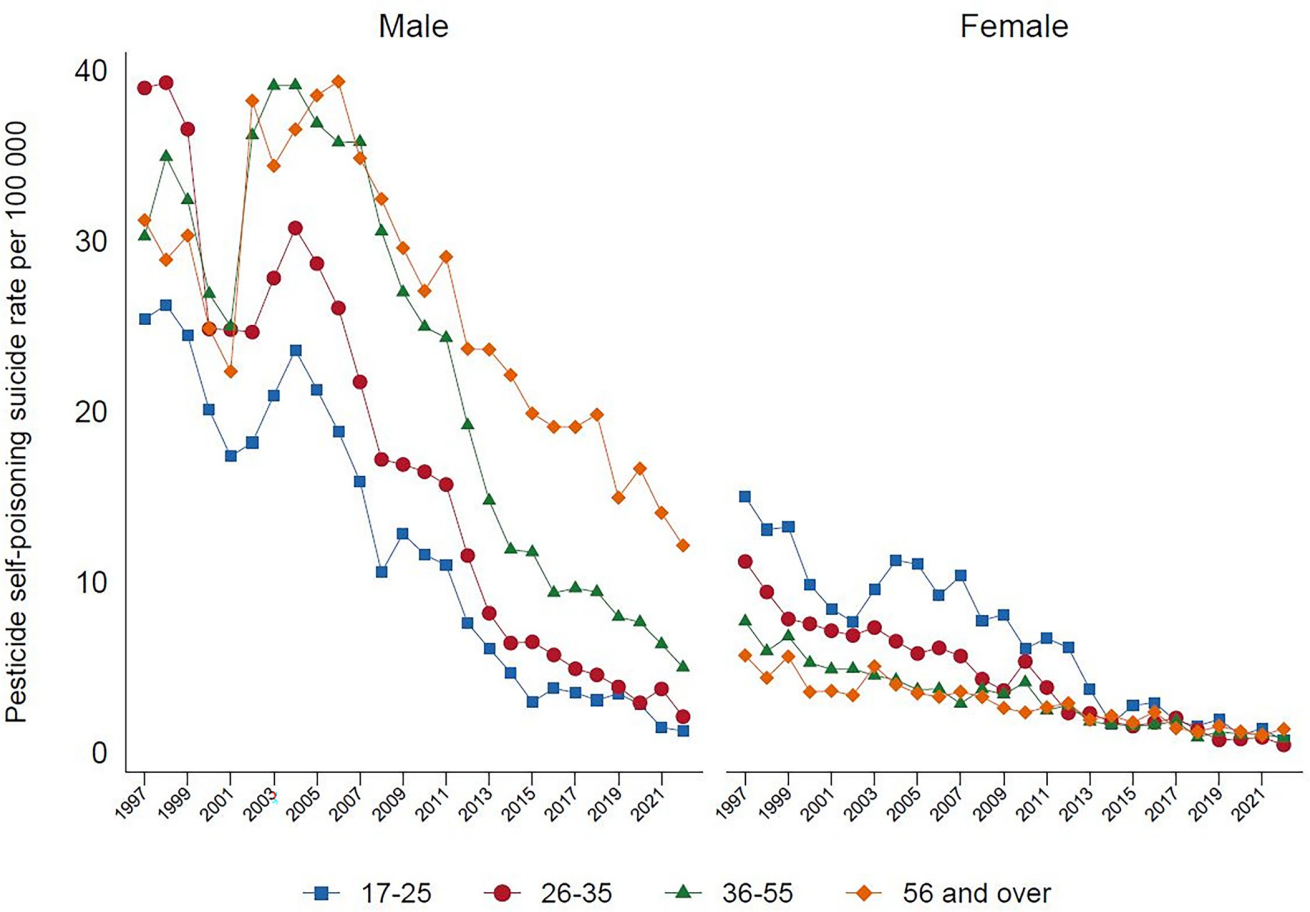
Pesticide self-poisoning suicide rate, by age and gender in Sri Lanka, 1997–2022.

**Fig 4:**
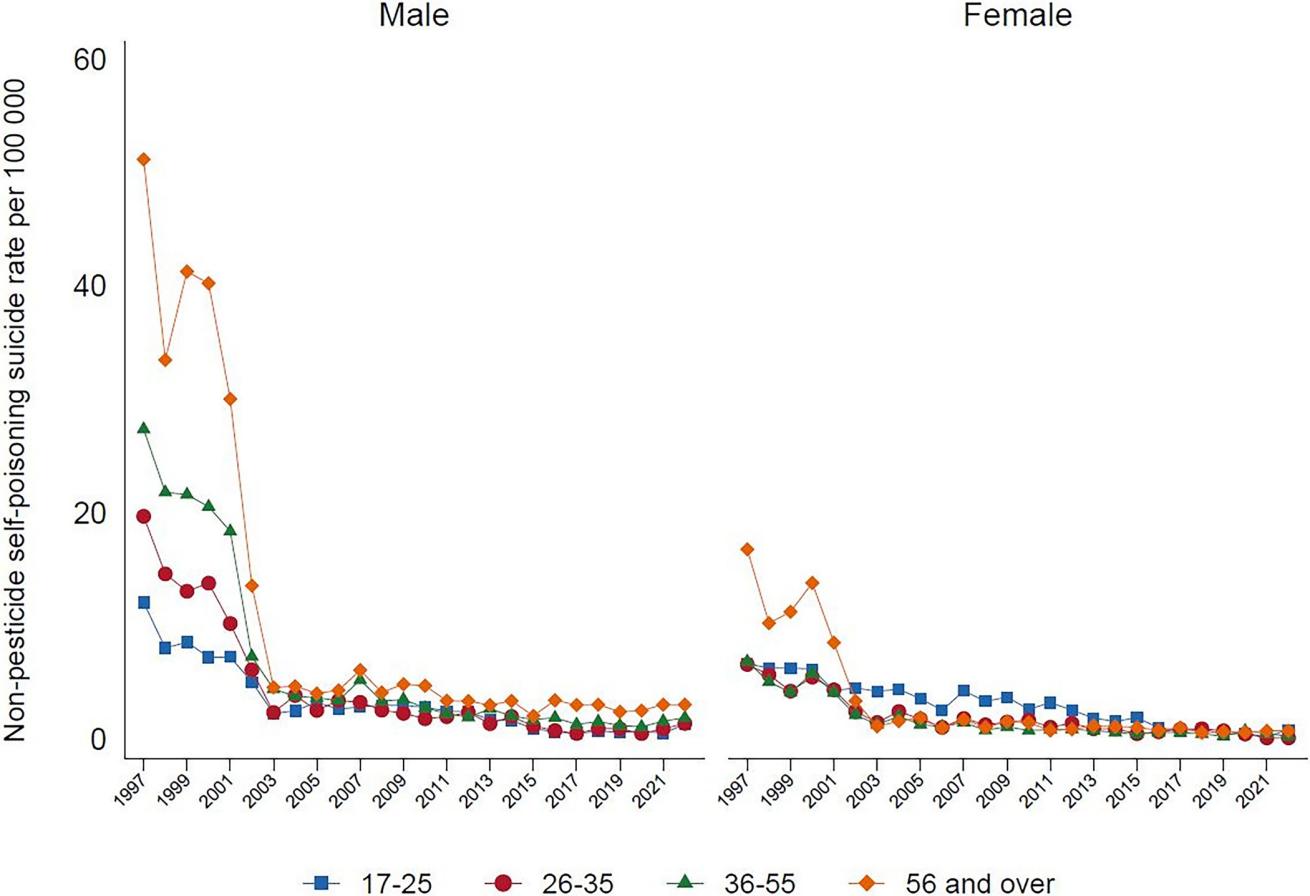
Non-pesticide self-poisoning suicide rates by age and gender in Sri Lanka, 1977–2022.

**Fig 5:**
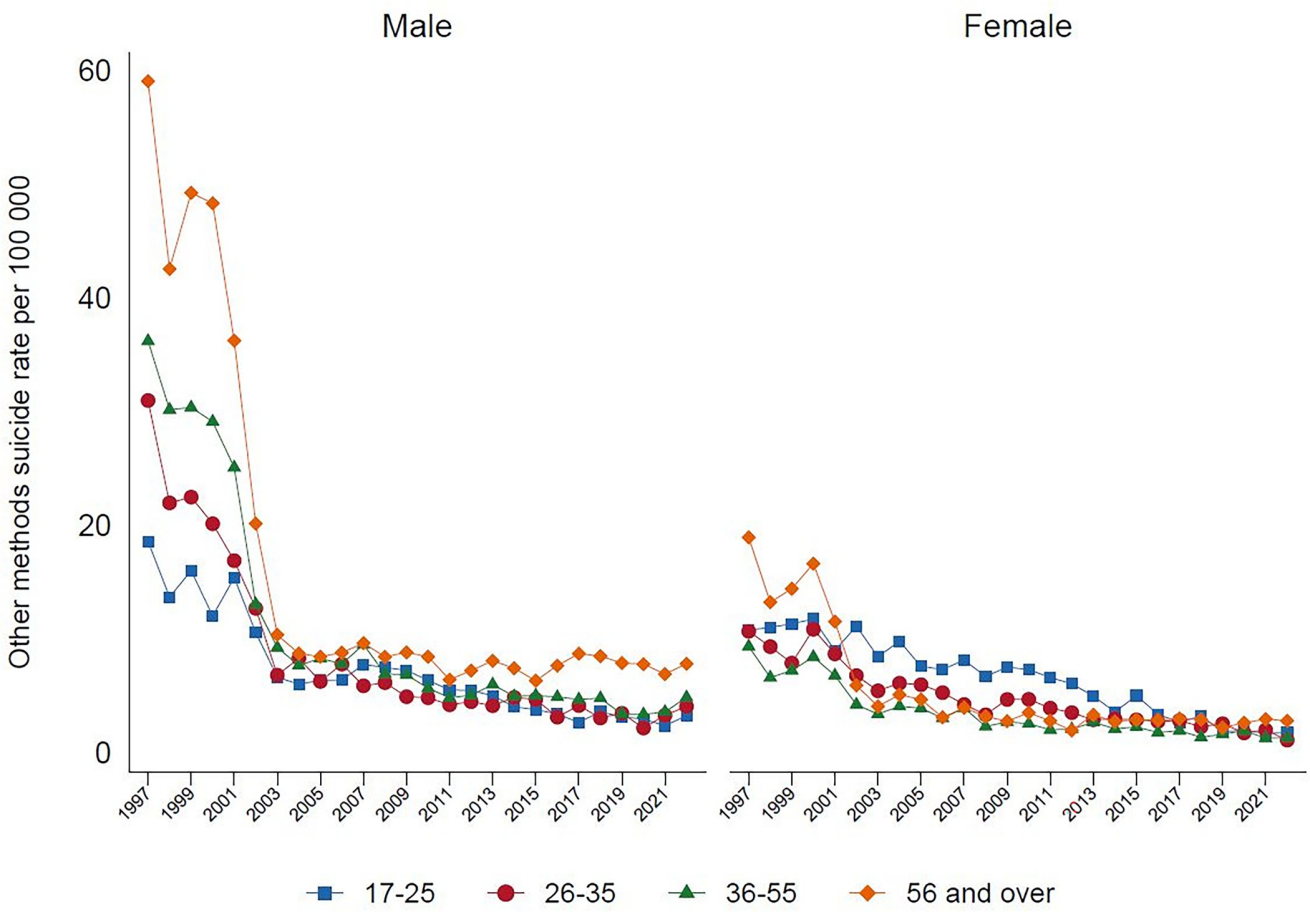
Rates of suicide by other methods, by age and gender in Sri Lanka, 1997–2022.

Analysis by method categorizations shows that in 2022, the highest proportion of suicide deaths were due to hanging (69.9%), followed by pesticide self-poisoning (14.0%), other methods (12.2%), and non-pesticide self-poisoning (3.9%). Suicide by hanging, over the past 20 years, has been steadily increasing among males and females. This is especially seen among males (Fig 1), with a marked increase amongst older males aged over 55 years-old (Fig 2). In females, the rise in hanging is most marked in the 17–25 year age group. At present hanging is the most common method of suicide in both sexes. There has been a parallel marked reduction in suicide by pesticide self-poisoning, and non-pesticide self-poisoning between 1997 to 2022, for males and females, and across all age groups (Figs 3, 4). Suicide rates by all other methods have declined since 1997 to 2022, with a sudden step change in 2002 (Fig 5) which reflects changes in the coding of suicide deaths at this time [1].

The overall F:M ratio is 1:5 in 2022 but this differs by age group, with narrower ratios in younger people (Table 1). Additionally, the ratio is narrower for pesticide self-poisoning and suicide by other methods, compared with hanging or non-pesticide poisoning.

## Discussion

Following decades of declining rates of pesticide-related suicides, from 2017 onwards Sri Lanka appears to be experiencing an upward trend in age-standardized male suicide rates due to hanging, especially amongst older men. During the previous two decades, the decline in overall suicide rates in Sri Lanka was driven largely by restriction of access to pesticides and consequent reductions in suicides due to pesticide ingestion [2]. The rates of suicide by pesticide ingestion are still declining, but the rate of suicide by hanging is rising. Despite this rise, the current rate of suicide is still significantly lower than prior to any national pesticide bans in the country. However, if the rate of hanging continues to rise, Sri Lanka’s overall suicide rate of 15 per 100,000 (which remains well above the global average of 9 per 100, 000) will likely increase [4]. Suicide rates by non-pesticide self-poisoning and other methods have declined since 1997 for males and females, with the sharpest reductions seen in 2002 which coincides with changes in the coding of suicide deaths [1].

The shifting patterns of increasing suicide by hanging are consistent with recent trends from other South Asian countries including India, Nepal and Bangladesh [16–19]. As these countries are becoming increasingly urbanized, this may reflect a change to methods of suicide traditionally more prevalent in high-income countries. Method substitution (i.e., hanging replacing ingestion of pesticides) following restricted access to highly hazardous pesticides, may partly explain this trend [20], however further evidence is needed.

With the rising rate of suicide by hanging and the consequent rise in overall suicide deaths in males, concerted attention is needed to monitor and address this rise, not only in Sri Lanka, but in South Asia in general. This is particularly so because as the world emerges from the shadow of the COVID-19 pandemic, many countries, and Sri Lanka in particular, are facing significant socio-economic challenges, with rapidly increasing inflation, cost of living, and unemployment rates [8, 21], which are also well-known risk factors for suicide, particularly among males [22, 23]. Previous evidence shows that a percentage rise in unemployment is associated with an 0.94% increase in suicide among males [22]. Problems such as financial difficulties, debts, and male unemployment go hand-in-hand with other challenges, such as increased domestic violence, alcohol misuse and depression–all of which increase the risk of suicide [24, 25]. Depression is still underrecognized in Sri Lanka, partly due to lack of awareness and the significant stigma still surrounding mental health problems [26]. In times of economic challenges, people are also less likely to seek help from healthcare services for psychological issues, and culturally may be less likely to view healthcare services as a possible source of support [27]. There is also the risk that governments will shift limited national finances away from mental health [28]. Measures to prevent an upward trend in suicides in this socio-economic context, is a challenge, but nonetheless, primary preventive efforts that address the underlying risk factors for suicide should be a key priority in these challenging times. Active labour market programs, provision of buffers for unemployment and increasing levels of social capital to enhance resilience in vulnerable groups may have efficacy in mitigating suicide risks during economic recessions and should be considered [22, 28]. Monitoring and regulation of loan schemes in the community, to minimize spiraling individual debt, should also be considered. Increasing awareness of mental health issues in the community, among primary care health professionals, in government and private work sectors, and strengthening national mental-health services, particularly preventive and rehabilitation services for alcohol and other substance misuse, should remain as a priority.

Although rates of suicide among females is lower than among males, a concerning finding is the high rate of suicide in young females, with narrower sex ratios in this age group. In females the rate of hanging suicide is greatest in young females aged 17–25 years, with suicide rates by hanging increasing over time. The finding of a higher rate of suicide in young females is similar to Sri Lanka’s neighbour India [29]. Domestic violence, gender inequality, and conflicts secondary to traditional expectations of women versus changing female gender roles, and difficulties in coping may all play a role in young female suicides [30–32]. Given these trends, it is imperative to consider whether suicide prevention interventions should also be tailored to meet the needs of varying sub-populations. This could be done with the involvement of appropriate local and national-level stakeholders.

The availability of lethal means is likely to increase risk of suicide. The World Health Organization recommends the banning of highly hazardous pesticides as a way in which to reduce suicide globally [33]. This approach is likely to save a number of lives worldwide. These restrictions need to be done in collaboration with a number of agencies. This should include agricultural, health and government authorities, but will need to include other partners too, most notably the media.

Means restriction of hanging in the general community is challenging given the easy access to ligature points and material, but due attention should be given to minimizing access to means as far as possible, especially in institutions such as prisons and hospital. A key aspect in prevention is to ensure that hanging doesn’t become publicly perceived as a method of choice, in countries where toxic pesticides become less available. Media portrayals of suicide are common in Sri Lanka, especially regarding suicide by hanging in recent years [34]. The risk of imitative suicides in response to sensationalized media reporting of suicide and suicide methods is well-established [35]. Inadvertent popularization of ‘hanging’ as a method of suicide, especially on social and online media, is a dangerous new trend, which is likely to promote suicide by this method. The media have a key role to ensure that, as the availability of one lethal method of suicide decreases, the cognitive availability of an equally lethal method of suicide doesn’t increase [36]. Ongoing dialogue is needed with media professionals regarding responsible reporting of suicide, which includes limiting disclosure of the suicide method, and promoting suicide prevention support and narratives of hope and resilience [34, 37, 38]. Implementation of an effective media policy, with the ownership of the media, could be a way forward. Innovative methods to address this issue via online platforms should be explored.

### Limitations

At the time of analysis, we did not have data by geographical district in Sri Lanka for each year, therefore, it was not possible to examine regional trends in suicide. Given that national bans on pesticide products are more likely to impact rural agricultural areas than urban areas, a regional analysis of suicide trends would provide more nuanced findings on potential shifts in method by area. Under-reporting is possible due to stigma and misclassification of suicide as shown in India [39].

## Conclusions

Despite significant reductions in suicide over the past two decades in Sri Lanka, the current suicide rate of 15 per 100,000 remains high and the increases in rates of hanging, especially among older males and younger females warrants further attention. Should the rise in hanging continue at its current rate, Sri Lanka’s overall suicide rate is likely to increase. Continued vigilance is needed. Interventions to address risk factors for suicide and self-harm that are likely to play a key role in the next few years, such as issues related to economic distress, unemployment, alcohol misuse, depression and domestic violence, and ongoing means restriction, as well as minimizing media glamourization of suicide, should be targeted as a priority, to maintain Sri Lanka’s progress in reducing its suicide rate.
